# The stem cell potential of bovine milk: a comparative study of colostrum and mature milk

**DOI:** 10.1007/s00418-026-02494-6

**Published:** 2026-05-25

**Authors:** Nurcan Delice, Serife Tutuncu, Melek Yuce

**Affiliations:** 1 Department of Histology and Embryology, Faculty of Veterinary Medicine, Ondokuz Mayıs University, Samsun, Turkey; 2 Department of Medical Services and Techniques, Health Services Vocational School, Ondokuz Mayıs University, Samsun, Turkey; 3 Stem Cell Research and Application Center, Ondokuz Mayıs University, Samsun, Turkey

**Keywords:** Bovine, Colostrum, Mature milk, Mesenchymal stem cell

## Abstract

Mesenchymal stem cells (MSCs) are widely utilized in regenerative medicine owing to their differentiation potential and paracrine effects. Although numerous tissues have been identified as sources of MSCs, the search for novel, noninvasive sources continues. To date, the stem cell content of colostrum has not been investigated. To address this gap, this study aimed to comparatively evaluate the biological properties, proliferative dynamics, and functional potential of cell populations derived from colostrum and mature milk. Cells isolated from both sources (*n* = 3) were morphologically assessed under in vitro culture conditions and were induced to undergo multilineage differentiation. Phenotypic characterization was performed by flow cytometric analysis. Proliferation capacity was assessed by determining population doubling time (PDT) and performing colony formation assays, while cell viability was evaluated using the methyl thiazolyl tetrazolium (MTT) assay. Cells initially exhibited an epithelial-like morphology and adopted a fibroblast-like phenotype after passaging. Colostrum-derived and mature milk-derived cells displayed multilineage differentiation potential, and flow cytometric profiling confirmed the presence of cells positive for CD73, CD90, and CD105 in both sources. Colostrum-derived cells exhibited higher cell densities; however, population doubling times showed no statistically significant difference between the groups. MTT analysis demonstrated a progressive increase in metabolic activity in both groups, with colostrum-derived cells exhibiting significantly higher optical density values from day 2 onward. No statistically significant difference in colony-forming efficiency was observed between the groups. Consequently, these preliminary findings suggest that colostrum may harbor cell populations exhibiting MSC-like properties and could represent a potential area of interest for MSC research.

**Graphical Abstract:**

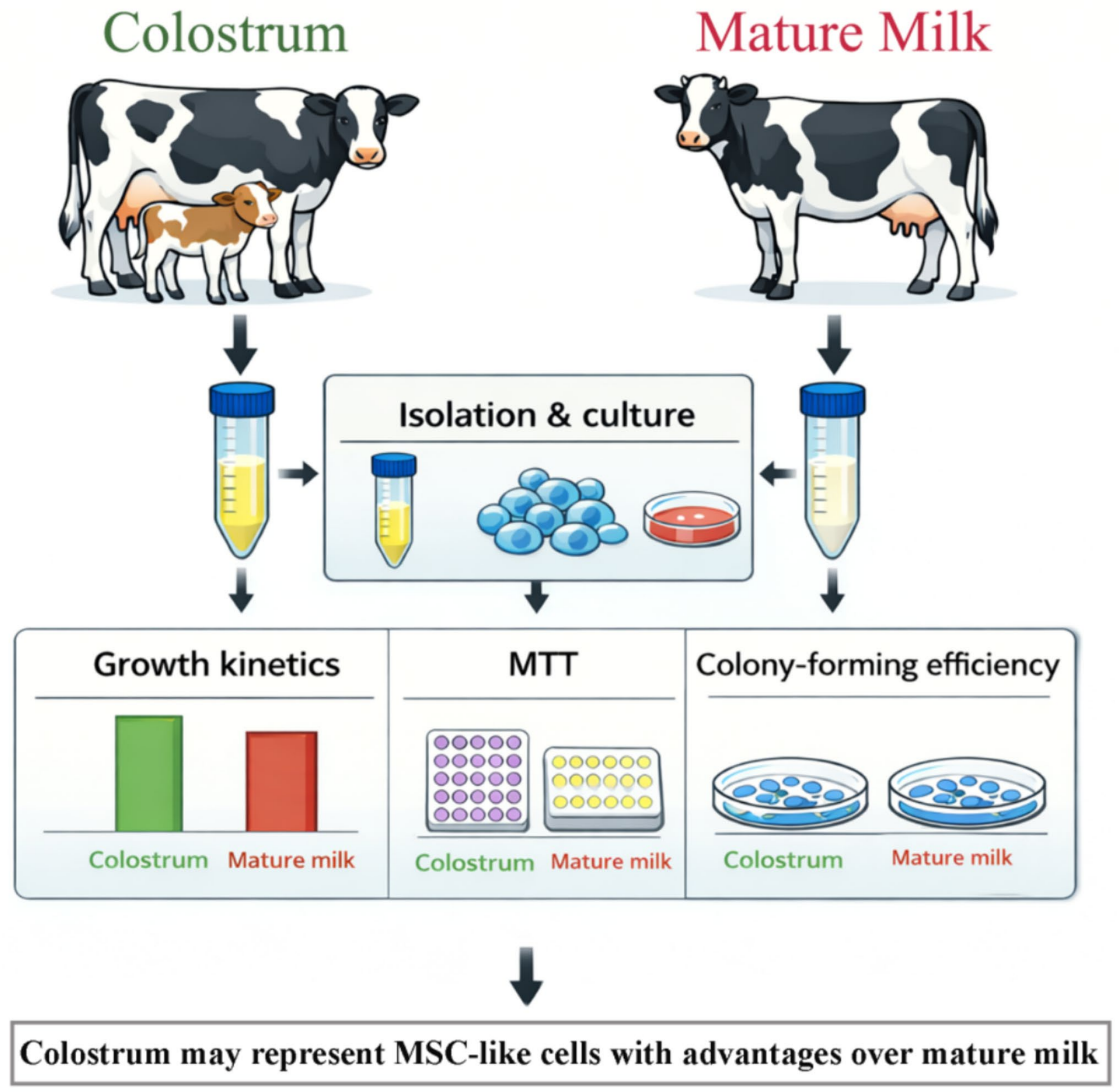

## Introduction

Stem cells are unspecialized cells that play a pivotal role in the development and maintenance of tissues and organs in an organism (Can 2014). Beyond their high differentiation potential, stem cells are paracrine-active entities that secrete numerous key growth factors, cytokines, trophic factors, and regenerative molecules (Özen and Gül Sancak 2014). Consequently, research in stem cell biology and stem cell-based therapies has gained significant attention in recent years. MSCs, a type of adult stem cell, are among the most widely used stem cells for therapeutic purposes. Their prominence is largely due to their accessibility from a wide range of sources, including bone, periosteum, peripheral blood, tendon, skeletal muscle, dental pulp, dermis, synovial fluid, adipose tissue, umbilical cord, hair follicle, cartilage, periodontal ligament, lung, spleen, endometrium, intervertebral disc, menstrual blood, pancreas, thymus, eye, retina, and fallopian tube (Frese et al. 2016; Özgenç 2019; Zomer et al. 2015). The emerging field of regenerative medicine has heightened the demand for novel MSC sources that can be obtained through noninvasive methods.

Milk is vital for mammalian nutrition, especially for neonates, as it provides high concentrations of essential components (Özek 2015). Colostrum, the first source of nutrition for neonatal mammals, is produced prepartum during late gestation and secreted postpartum, primarily during the first 5 days (Suzan 2020; Cao et al. 2019). The term “mature milk” denotes breast milk secreted beginning around the 15th day postpartum and continuing for the duration of lactation (Suzan 2020). Colostrum is richer than mature milk in bioactive components, particularly immunoglobulins, antimicrobial peptides, and growth factors. It is an indispensable natural source of nutrients for the growth, development, and immunological defense of newborn calves (Koyuncu and Karaca 2018). Beyond its role in promoting the health of newborn calves, colostrum is also a valuable supplement in human nutrition (Besser and Gay 1994). Bovine colostrum, with a history of use in human nutrition stretching back millennia, was employed as an antibacterial agent before the development of antibiotics. The 1990s also saw a significant rise in scientific interest regarding the application of bovine colostrum in human nutrition and the formulation of related products (Jenny et al. 2010). Despite extensive literature searches, no studies on the isolation of stem cells from bovine colostrum have been reported. Investigations into stem cells derived from mature milk are limited to a single study by Pipino et al. (2018).

The principal objective of this study was to compare the key biological properties of cell populations isolated from bovine colostrum and mature milk, focusing on their growth, proliferation, multilineage differentiation, and surface marker expression. In this context, the study was designed to provide preliminary insights into the potential of colostrum as a novel, noninvasive source of MSC-like cells and to contribute to the limited body of knowledge on bovine mature milk-derived cells.

## Materials and methods

### Collection of bovine milk samples

Bovine milk samples were obtained during routine milking from dairy farms in the Bafra region of Samsun. Colostrum samples (*n* = 3) were collected within 24 h postpartum, whereas mature milk samples (*n* = 3) were collected from bovines 30–35 weeks postpartum (Cao et al. 2019; Fadlalla et al. 2020). The number of animals included in the study was determined using a power analysis (Tang et al. 2019). After discarding the foremilk, 100 mL of milk was aseptically collected directly into sterile containers containing 100 mL of phosphate-buffered saline (PBS; Sigma-Aldrich, St. Louis, MO, USA) presupplemented with 5% penicillin–streptomycin (Sigma-Aldrich, St. Louis, MO, USA) and 5% amphotericin B (Sigma-Aldrich, St. Louis, MO, USA). This procedure ensured a 1:1 (vol/vol) ratio between the milk and the protective buffer at the time of sampling. All samples were transported to the laboratory under aseptic conditions.

### Isolation and culture of bovine milk-derived cells

Bovine milk was centrifuged at 228*g* for 15 min at room temperature (Nuve, Ankara, Turkey). The fat layer and supernatant were discarded, and the cell pellet was washed with sterile PBS containing 1% penicillin–streptomycin and 1% amphotericin B (Gibco, Thermo Fisher Scientific, Grand Island, NY, USA). The cells were then re-centrifuged at 400*g* for 5 min to obtain a clean pellet. Finally, the cells were resuspended in a standard culture medium composed of minimum essential medium alpha (α-MEM; Sigma-Aldrich, St. Louis, MO, USA) supplemented with 10% fetal bovine serum (FBS; Gibco, Thermo Fisher Scientific, Grand Island, NY, USA), 1% penicillin–streptomycin, and 1% l-glutamine (Capricorn Scientific GmbH, Germany). The cells were then cultured in an incubator at 37 °C and 5% CO_2_ (Heracell 150i, Thermo Scientific, Grand Island, NY, USA). The culture medium was replaced with fresh medium every 2 days. For subculturing and expansion, cells were detached using 0.25% trypsin–EDTA (Sigma-Aldrich, St. Louis, MO, USA) upon reaching 80–90% confluence. All experimental assays were performed using cells between passages 3 and 4 (Pipino et al. 2018).

### Multipotency assays

To assess osteogenic differentiation, passage 3 cells were cultured in an osteogenic medium after they reached approximately 80% confluence and the previous culture medium was removed. The osteogenic medium consisted of low-glucose Dulbecco’s modified Eagle medium (DMEM-LG) supplemented with 0.05 mM ascorbic acid-2-phosphate (Sigma-Aldrich, St. Louis, MO, USA), 10 mM β-glycerophosphate (Sigma-Aldrich, St. Louis, MO, USA), and 100 nM dexamethasone (Sigma-Aldrich, St. Louis, MO, USA). Additionally, the medium included 10% FBS, 1% penicillin–streptomycin, and 1% l-glutamine (Pipino et al. 2018). The medium was refreshed every 2 days, and the cells were cultured for 21 days. At the end of the culture period, cells were fixed in 4% paraformaldehyde. After fixation, cells were stained with 2% Alizarin Red S solution (pH 4.2; Sigma-Aldrich, St. Louis, MO, USA) for 1 h (Im et al. 2005).

To induce chondrogenic differentiation, milk-derived cells were incubated for 21 days in high-glucose Dulbecco’s modified Eagle medium (DMEM-HG) supplemented with 1% FBS, 1% penicillin–streptomycin, 1% l-glutamine, 100 nM dexamethasone, 1% insulin–transferrin–selenium–ethanolamine (ITS-X; Invitrogen, Carlsbad, CA, USA), 50 μg/mL ascorbate-2-phosphate, 1 mM sodium pyruvate (Sigma-Aldrich, St. Louis, MO, USA), and 10 ng/mL TGF-β1 (Sigma-Aldrich, St. Louis, MO, USA) (Pipino et al. 2018). Following incubation, cells were fixed in 4% paraformaldehyde and stained with 1% Alcian blue solution (Isolab, Eschau, Germany) (Murata et al. 2014).

For adipogenic differentiation, cells were cultured for 21 days in DMEM-HG-based adipogenic medium supplemented with 10% FBS, 1% penicillin–streptomycin, 1% l-glutamine, 1 mM dexamethasone (Sigma-Aldrich, St. Louis, MO, USA), 0.5 mM 3-isobutyl-1-methylxanthine (Sigma-Aldrich, St. Louis, MO, USA), 100 μM indomethacin (Sigma-Aldrich, St. Louis, MO, USA), and 10 μg/mL insulin (Gibco, Thermo Fisher Scientific, Shah Alam, Malaysia) (Ghorbani et al. 2014). The medium was refreshed every 2 days. Following differentiation, cells were fixed in 4% paraformaldehyde (Sigma-Aldrich, St. Louis, MO, USA) and stained with Oil Red O (Grimstone and Skaer 1972). Histological images were acquired using a Nikon Eclipse E600 research microscope equipped with a Digital Sight DS-L1 camera system (Nikon Corporation, Tokyo, Japan).

### Flow cytometry

Passage-3 cells were characterized for specific cell-surface antigens by flow cytometry (BD FACSCanto™ II Cell Analyzer, San Jose, CA, USA). A total of 1 × 10^5^ cells were suspended in 100 μL of PBS supplemented with 2% FBS and incubated at room temperature for 30 min with FITC-conjugated anti-CD90 (BioLegend, San Diego, California, USA, cat. no. 328108), PerCP/Cyanine5.5-conjugated anti-CD105 (BD Pharmingen, San Diego, CA, USA, cat. no. 560819), and APC-conjugated anti-CD73 (BioLegend, San Diego, California, USA, cat. no. 344006) monoclonal antibodies. Antibodies were used according to the manufacturer’s instructions (5 µL antibody per 10^6^ cells in 100 µL staining volume). Antibodies were purchased from BioLegend and BD and were validated by the manufacturers for flow cytometry applications. Specificity was confirmed on the basis of the manufacturers’ technical data sheets. After incubation, cells were centrifuged at 400^g^ for 5 min; the supernatant was discarded, and the cells were washed with PBS. The cells were then resuspended in 200 μL of PBS, and at least 2 × 10^4^ cells were analyzed by flow cytometry (Teshima et al. 2020; Teunissen et al. 2021).

### Determination of growth curve and population doubling time (PDT)

To compare the growth kinetics of cells from different sources, growth curves based on cell counts were plotted, and the PDT was determined. Cells were seeded in 6-well culture plates (TPP, Trasadingen, Switzerland) at an initial density of 5 × 10^4^ cells per well. The cells were harvested by trypsinization on seven consecutive days. After trypan blue staining, cells were counted manually using a hemocytometer. The PDT was calculated using the following formula: PDT = (*t* − *t*_0_) × log_2_ × (log < *N*/*N*_0_ >)^−1^, where *t* is time, *t*_0_ is the initial time, *N* is the number of cells, and *N*_0_ is the initial number of cells. All experiments were performed in triplicate for each group, and the mean values were calculated (Yang et al. 2018).

### MTT assay

To further evaluate cellular proliferation and metabolic activity, an MTT assay was performed. Cells were seeded in 96-well plates at a density of 2 × 10^3^ cells per well. Cell proliferation was monitored over a 7-day period, with measurements taken at daily intervals. For each time point, 10 μL of 3-(4,5-dimethylthiazol-2-yl)-2,5-diphenyl tetrazolium bromide (5 mg/mL MTT, Serva, Heidelberg, Germany) was added to each well, which was then incubated at 37 °C for 4 h. Following incubation, 100 μL of solubilization solution (10% SDS prepared in 0.01 M HCl) was added to each well, and the plates were incubated for an additional 16 h. The absorbance was then measured at 570 nm using a microplate reader (Thermo Scientific™ Multiskan™ GO Microplate Spectrophotometer, Vantaa, Finland). All experiments were performed in triplicate for each group (Wu et al. 2018).

### Colony-forming unit assay

A clonogenic assay was performed to assess the colony-forming potential of the cells. Passage 3 cells were seeded into 6-well plates at a low density of 100 cells per well in triplicate. Following a 2-week incubation period, the resulting colonies were fixed with 4% paraformaldehyde for 10 min and stained with 0.3% (vol/vol) crystal violet for 5 min. Only colonies consisting of more than 50 cells were counted. The colony-forming efficiency (CFE) was then calculated as follows: CFE (%) = (number of colonies counted/number of cells seeded) × 100 (Pereira et al. 2012).

### Statistical analyses

All data were analyzed using the Statistical Package for the Social Sciences (SPSS) version 22 for Windows. Group comparisons were performed using Student’s *t*-test and the Mann–Whitney *U* test. A *p*-value of less than 0.05 was considered statistically significant.

## Results

### Morphology of bovine milk-derived cells

**Fig. 1:**
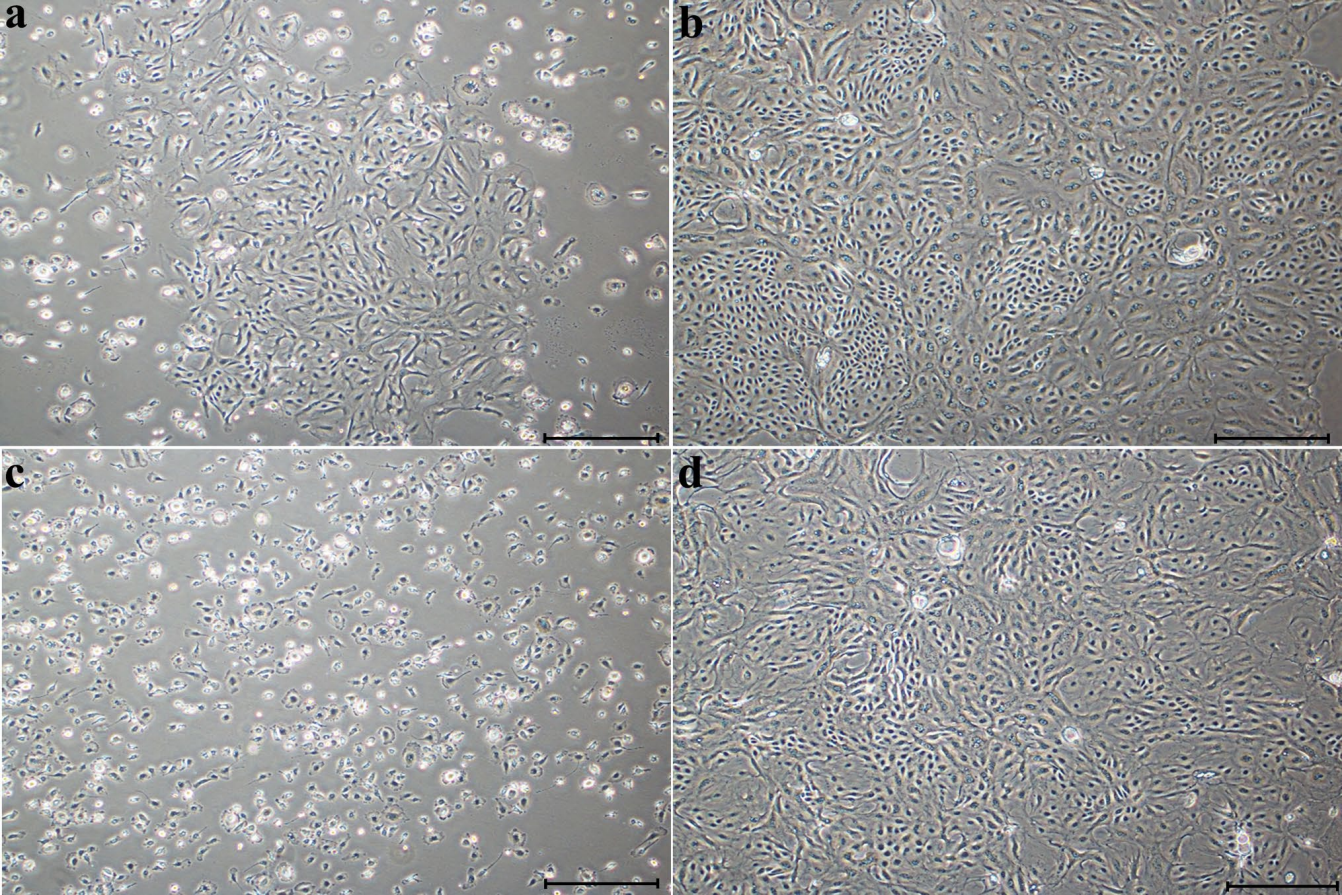
Morphology of cells from colostrum and mature milk at different passage stages. **a** Colostrum, P0; **b** colostrum, P2; **c** mature milk, P0; **d** mature milk, P2. Scale bar: 100 µm

Cells isolated from bovine colostrum and mature milk initially exhibited a dispersed morphology in culture, but they adhered to the culture surface within 24–48 h. These adherent cells primarily exhibited a round or polyhedral epithelial-like morphology; a subset of cells contained visible cytoplasmic vacuoles. After two passages, the majority of the cell population transitioned to a spindle-shaped, fibroblast-like phenotype (Fig. 1).

### The multilineage potential of bovine milk-derived cells

**Fig. 2:**
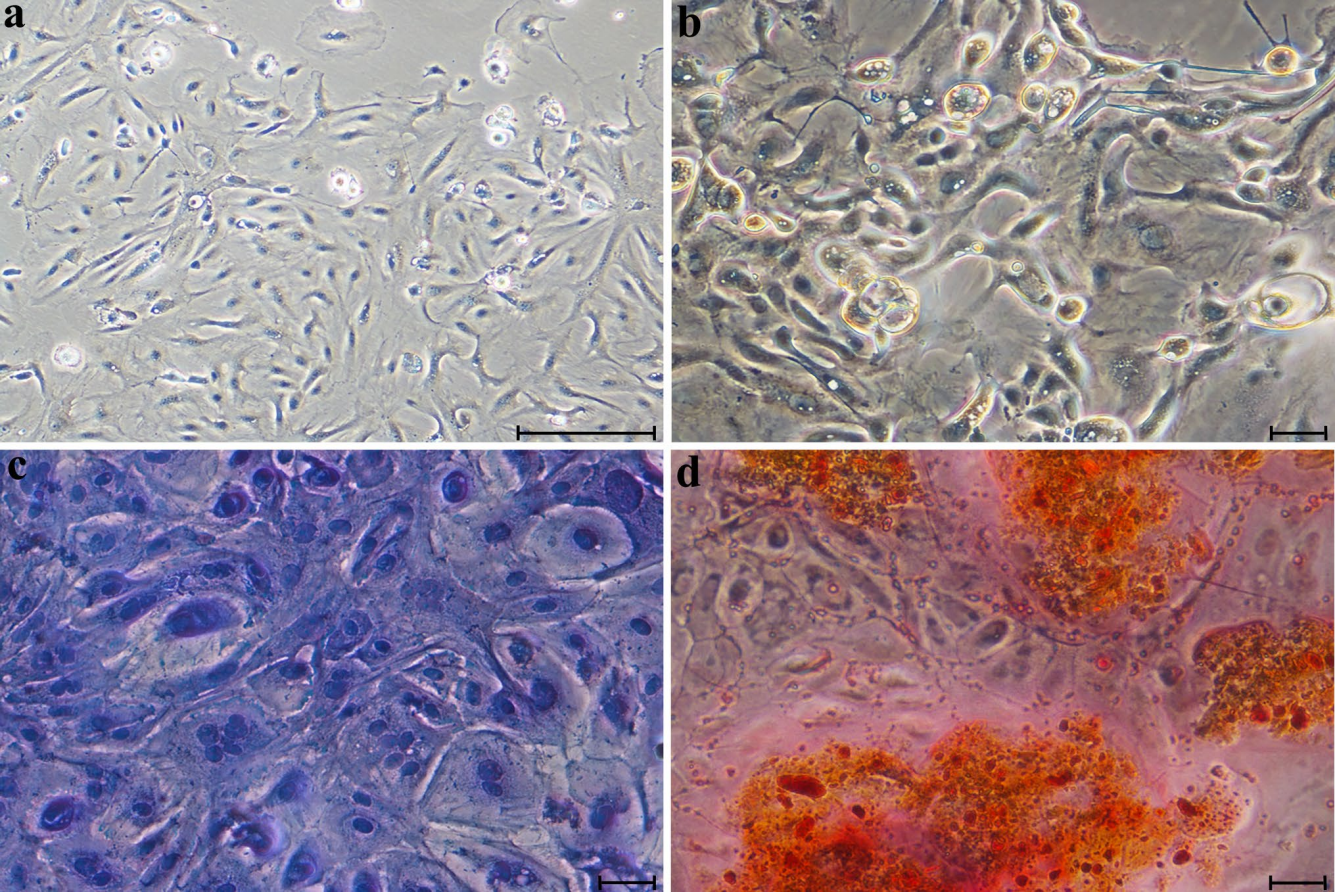
Differentiation of colostrum-derived cells into adipogenic (**b**), chondrogenic (**c**), and osteogenic (**d**) lineages; cells maintained under standard culture conditions are presented in panel (**a**). Scale bars: 50 µm (**a**), 10 µm (**b–d**)

**Fig. 3:**
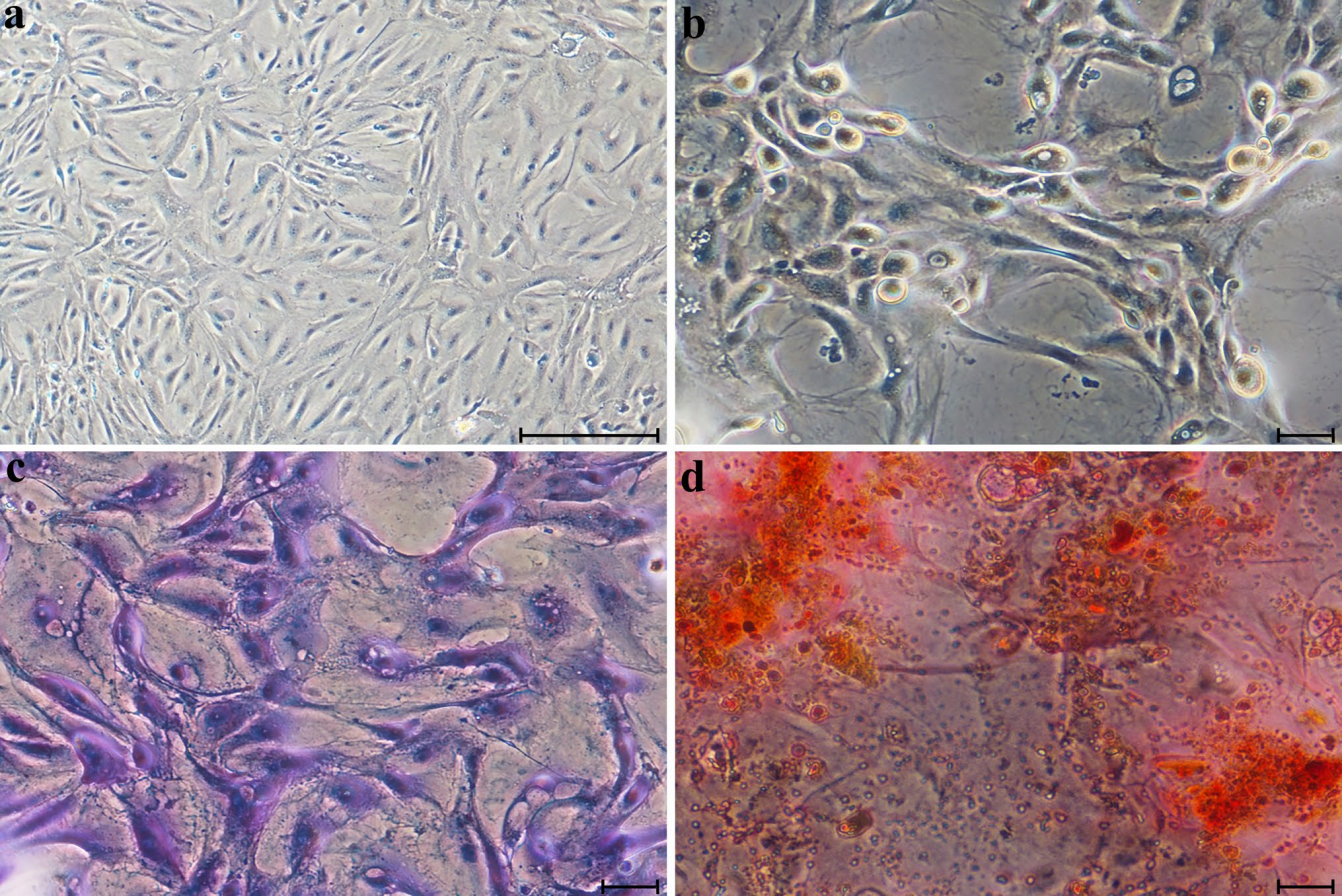
Differentiation of mature milk-derived cells into adipogenic (**b**), chondrogenic (**c**), and osteogenic (**d**) lineages; cells maintained under standard culture conditions are presented in panel (**a**). Scale bars: 50 µm (**a**), 10 µm (**b–d**)

Following adipogenic induction, microscopic examination revealed that the cells had differentiated into adipocytes, as demonstrated by the presence of lipid droplets in their cytoplasm. Oil Red O staining confirmed the differentiation, with the lipid droplet clusters appearing red. Chondrogenic differentiation produced an extracellular matrix rich in glycosaminoglycans and proteoglycans, as confirmed by Alcian blue staining. After induction with osteogenic medium, a marked increase in cell proliferation was observed, followed by the formation of nodular aggregates and calcium deposition, as confirmed by Alizarin Red staining. These findings suggest that cells isolated from bovine colostrum and mature milk exhibit multipotency, as evidenced by their capacity to differentiate into adipogenic, chondrogenic, and osteogenic lineages (Figs. 2, 3).

### Surface marker profiling of bovine milk-derived cells

**Fig. 4:**
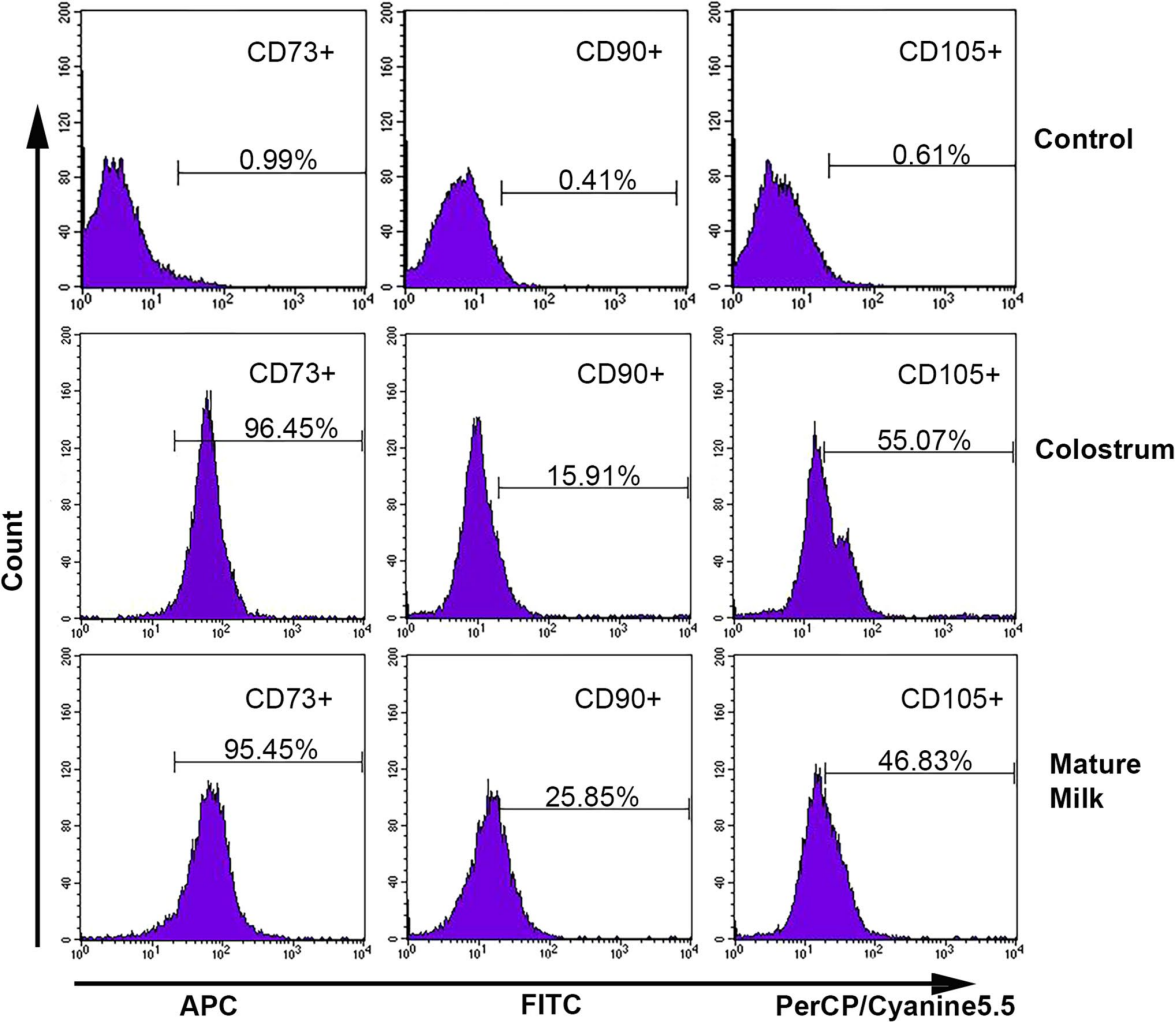
Representative histograms showing the expression of MSC associated markers CD73, CD90, and CD105 in passage-3 colostrum-derived and mature milk-derived cells. Percentages of positive cells are indicated within each histogram

The immunophenotypic profile of passage 3 bovine milk-derived cells was analyzed by flow cytometry. Flow cytometric analysis revealed the presence of cell populations expressing common MSC surface markers in both cell sources. As presented in Table 1, CD73 was highly expressed in both colostrum- and mature milk-derived populations, with mean percentages being comparable in both groups (96.45% ± 1.15 versus 95.45% ± 0.54, respectively; *p* = 0.243). Both cell sources exhibited a lower frequency of positivity for CD90 and CD105 than for CD73. The mean percentage of CD90^+^ cells in the mature milk group was significantly higher (25.85% ± 1.89) than that of the colostrum group (15.91% ± 4.36; *p* = 0.022). Importantly, no statistically significant difference in CD105 expression was observed between colostrum- and mature milk-derived cells (*p* = 0.581). Representative flow cytometry histograms illustrating these expression patterns are shown in Fig. 4.

**Table 1:** Expression of MSC surface markers in colostrum-derived and mature milk-derived cells

Surface marker	Colostrum (% positive cell, mean ± SD)	Mature milk (% positive cell, mean ± SD)	*p*-Value
CD73	96.45 ± 1.15	95.45 ± 0.54	0.243
CD90	15.91 ± 4.36	25.85 ± 1.89*	0.022
CD105	55.07 ± 21.75	46.83 ± 3.43	0.581

*p* < 0.05; *statistically significant

### Proliferation capacity of bovine milk-derived cells

**Fig. 5:**
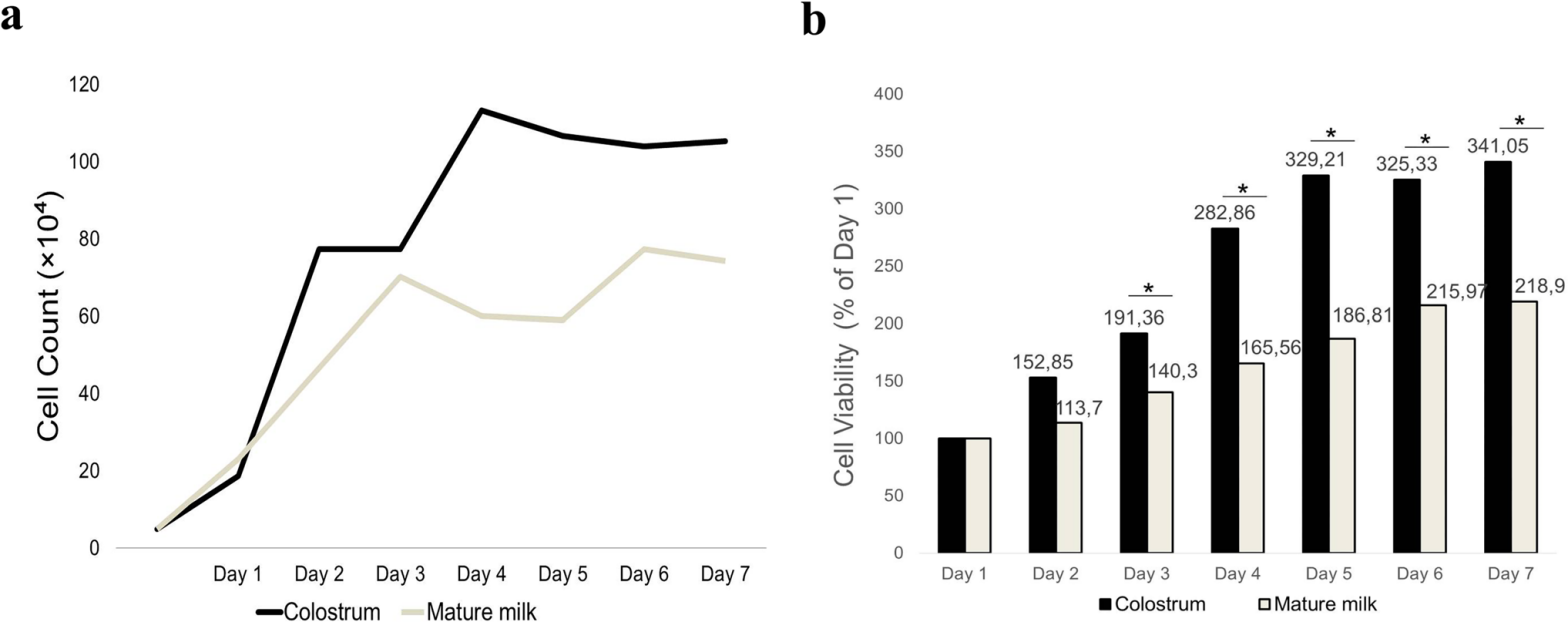
**a** Changes in the total cell count of colostrum- and mature milk-derived cells over a 7-day culture period, **b** Comparison of cell viability of colostrum-derived and mature milk-derived cells over 7 days using the MTT assay. Colostrum-derived cells displayed significantly higher viability on days 2, 3, 4, 5, and 7. *p* < 0.05; *statistically significant

The proliferation kinetics of cells derived from colostrum and mature milk were monitored over a 7-day period. During the first 24 h, both cell types exhibited a similar proliferation pattern. From day 2 onward, cell density increased more prominently in the colostrum group than in the mature milk group, reaching a higher peak density by the end of the culture period (Fig. 5a).

Cellular metabolic activity, as an indicator of cell viability, was assessed using the MTT assay. MTT absorbance values were normalized to day 1 (100%), and colostrum-derived cells reached 341.05% by day 7, whereas mature milk-derived cells reached 218.90% (Fig. 5b). Statistical analysis indicated that colostrum group exhibited significantly higher metabolic activity than mature milk group from day 2 onward (*p* < 0.05, Table 2).

**Table 2:** Optical density values of colostrum and mature milk groups at different days

Days	Colostrum (mean ± SD)	Mature milk (mean ± SD)	*p*-Value
1	0.188 ± 0.004	0.231 ± 0.035	0.08
2	0.286 ± 0.004^*^	0.262 ± 0.005	0.02
3	0.358 ± 0.016^*^	0.324 ± 0.011	0.02
4	0.530 ± 0.010^*^	0.382 ± 0.013	0.02
5	0.616 ± 0.036^*^	0.431 ± 0.021	0.02
6	0.609 ± 0.015^*^	0.498 ± 0.026	0.02
7	0.638 ± 0.008^*^	0.505 ± 0.013	0.02

Data were analyzed using the Mann–Whitney *U* test. Values are presented as mean ± standard deviation

*p* < 0.05; *statistically significant

Despite these differences, population doubling time (PDT) analysis did not reveal any statistically significant differences between the two groups at any time point (*p* > 0.05; Table 3). This discrepancy between metabolic activity and PDT values may suggest that the observed differences are associated with variations in cellular metabolic activity rather than intrinsic proliferation rates. Alternatively, it may reflect differences in cell size, mitochondrial activity, or assay sensitivity.

**Table 3:** Population doubling times of colostrum-derived and mature milk-derived cells

Days	Colostrum (mean ± SD)	Mature milk (mean ± SD)	*p*-Value
1	0.54 ± 0.08	0.47 ± 0.11	0.369
2	0.56 ± 0.17	0.63 ± 0.06	0.507
3	0.79 ± 0.12	0.80 ± 0.07	0.827
4	0.89 ± 0.04	1.13 ± 0.10	0.050
5	1.14 ± 0.08	1.45 ± 0.22	0.077
6	1.38 ± 0.06	1.53 ± 0.12	0.077
7	1.60 ± 0.06	1.82 ± 0.14	0.116

### Colony formation efficiency

**Fig. 6:**
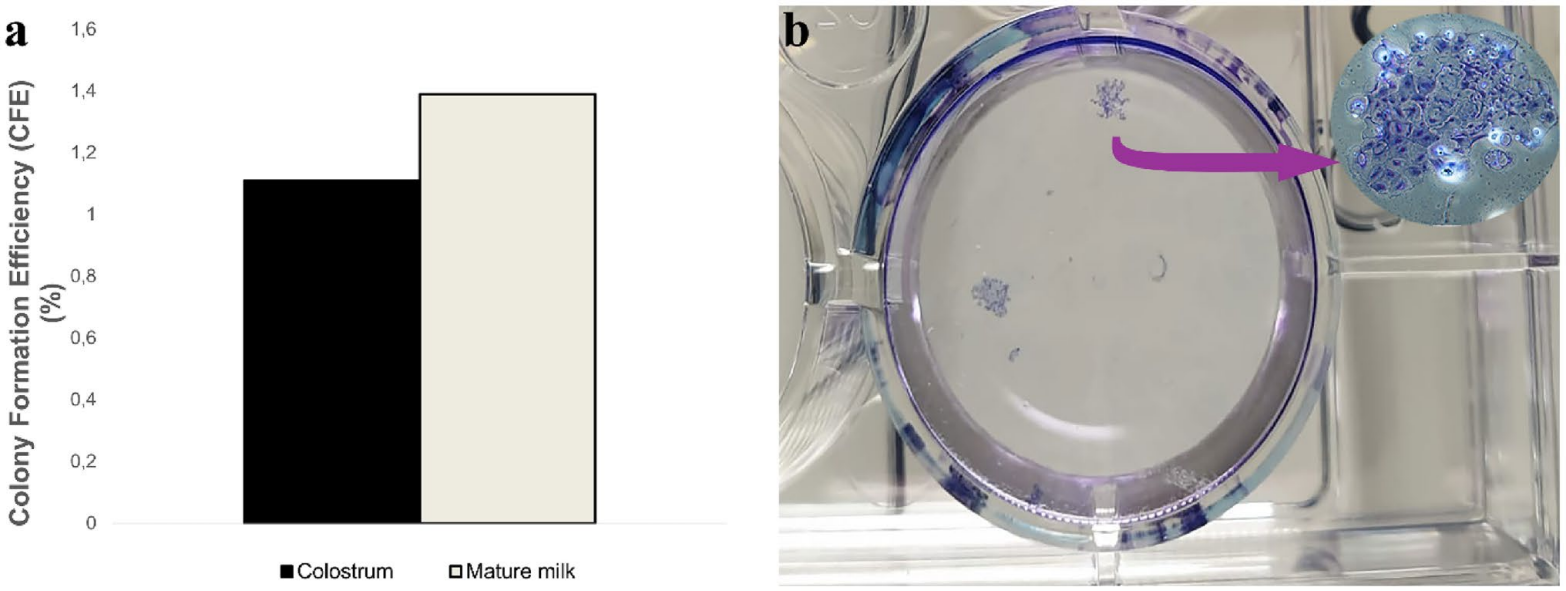
CFE of colostrum-derived and mature milk-derived cells. **a** A quantitative comparison revealed no statistically significant difference in CFE between the two groups (*p* > 0.05). A representative image of the colonies, stained with crystal violet after 14 days in culture, is shown in panel (**b**). The pink arrow indicates the microscopic morphology of a typical colony observed under a 4× objective

Colonies formed by passage-3 cells isolated from colostrum and mature milk were counted after 14 days in culture. The mean colony numbers were 1.11 ± 1.82 and 1.39 ± 1.32 for colostrum-derived and mature milk-derived cells, respectively (Fig. 6). No significant difference was observed between the groups (*p* > 0.05).

## Discussion

MSCs, initially isolated from bone marrow, have subsequently been identified in a wide range of adult tissues with similar phenotypic and functional characteristics (Berebichez-Fridman and Montero-Olvera 2018; Mafi et al. 2011). Bovine milk, however, remains a largely unexplored and noninvasive source of stem cells. The presence of stem/progenitor cells in milk was first demonstrated in human breast milk by Cregan et al. (2007). Patki et al. (2010) proposed that human breast milk contains MSCs and suggested that these cells may originate from myoepithelial cells. Dutta and Burlingham (2010) proposed that maternal cells are transferred to the offspring’s liver via breast milk. The presence of pluripotent stem cells in human milk was first reported by Hassiotou et al. (2012). In contrast, the stem cell population in bovine milk was not investigated until 2018, when Pipino et al. published the first study on the topic (Pipino et al. 2018). A comprehensive review of the literature revealed that no studies have yet examined the stem cell content of bovine colostrum, highlighting a significant gap in the field.

Equally important to identifying a biological material as a source of stem cells is understanding the fundamental cellular characteristics of cells derived from that source. In our study, the cultured cells exhibited adherent growth, attached to the culture surface, and displayed a typically elongated, spindle-shaped, fibroblast-like morphology characteristic of MSCs as defined by the International Society for Cellular therapy (ISCT) (Dominici et al. 2006). This fibroblast-like morphology is a hallmark of MSCs and has been consistently observed in cells isolated from a diverse range of species and tissue sources (Yang et al. 2018; Maldonado et al. 2024; Xiong et al. 2014; Sung et al. 2008). Although adherence has been reported in cells derived from human colostrum, this property was not observed in cells from mature human milk (Goudarzi et al. 2020). However, stem cells isolated from bovine milk by Pipino and colleagues exhibited plastic adherence, consistent with our findings, and presented a heterogeneous population of round and spindle-shaped cells (Pipino et al. 2018). Another key characteristic of MSCs is their ability to differentiate into at least three mesodermal lineages (Hocking and Gibran 2010). In our study, cells derived from colostrum and mature bovine milk demonstrated successful adipogenic, osteogenic, and chondrogenic differentiation when exposed to appropriate induction media. These findings are consistent with results from multilineage differentiation assays reported in other studies involving MSCs (Al-Nbaheen et al. 2013; Maldonado et al. 2024; Patki et al. 2010; Pipino et al. 2018). Numerous studies have investigated the surface marker characterization of MSCs. The most frequently reported positive markers include CD105, CD90, CD44, CD73, CD29, CD13, CD146, CD54, and CD166, while the most commonly reported negative markers include CD34, CD14, CD45, CD11b, CD49d, CD10, and CD31. Additionally, a variety of other cell surface markers have been described, including STRO-1, SH2, SH3, SH4, HLA-A, HLA-B, HLA-C, HLA-DR, HLA-I, DP, EMA, DQ (MHC class II), Oct4, Oct4A, Nanog, Sox-2, TERT, Stat-3, fibroblast surface antigen, α-SMA, vimentin, integrin subunits alpha4, alpha5, and beta1; integrins alpha-v beta3 and alpha-v beta5; and ICAM-1 (Mafi et al. 2011). According to the criteria established by the ISCT, a cell population must exhibit over 95% positivity for CD73, CD90, and CD105 to be defined as MSCs (Horwitz et al. 2005). In our study, the surface antigen profiles of cells derived from colostrum and mature milk showed that only CD73 expression (96.45% ± 1.15 and 95.45% ± 0.54, respectively) exceeded the ISCT-defined threshold. In contrast, the levels of CD90 and CD105 remained below the 95% requirement. Specifically, CD90 expression was low in both groups, although it increased significantly from colostrum (15.91% ± 4.36) to mature milk (25.85% ± 1.89; *p* = 0.022). This difference may be associated with changes in the lactogenic microenvironment during lactation. A study by Briere et al. (2017) revealed a marked divergence in the surface marker expression of breast milk cells from mothers who delivered prematurely compared with those who delivered at term, thereby supporting the likelihood of donor-related variation in the phenotypic characteristics of these cells. Regarding CD105, expression was moderate (55.07% ± 21.75 and 46.83% ± 3.43, respectively) without reaching the ISCT-defined threshold. Taken together, these findings indicate that bovine milk-derived cells do not conform to the classical MSC definition but instead represent a phenotypically heterogeneous MSC-like population. This heterogeneity may be associated with changes in the lactogenic microenvironment during lactation.

Our observations regarding phenotypic heterogeneity are consistent with previous reports on milk-derived cellular populations. Pipino et al. (2018) reported that only 30–40% of cells isolated from bovine milk expressed classical MSC markers, including CD90, CD73, and CD105. Indumathi et al. (2013) found that human milk-derived cells showed minimal expression of CD90 (7.7% ± 0.8) and CD73 (2.1% ± 0.41), whereas CD105 was expressed at 47.7% ± 2.95. Our findings are congruent with a larger study demonstrating that human milk-derived cells exhibit heterogeneous expression patterns of MSC markers. That study demonstrated expression of MSC-associated markers, including CD44 (88.3% ± 4.3), CD90 (41.6% ± 0.4), CD271 (78.8% ± 5.8), and CD146 (43.8% ± 5.8). In contrast, markers such as CD73 (3.8% ± 0.51) and CD105 (2.64% ± 0.55) were observed at markedly low levels (Sani et al. 2015). These findings suggest that bovine milk-derived cells constitute a phenotypically heterogeneous population. Importantly, it should be noted that ISCT criteria were originally established for human bone marrow-derived MSCs (Horwitz et al. 2005) and may not be directly applicable to cells derived from nontraditional sources or different species. As such, these criteria may require refinement to accommodate the biological variability observed in milk-derived stem cell populations. In addition, the relatively low expression levels observed for CD90 and CD105 may not solely reflect biological characteristics but could also be influenced by technical factors. Variations in antibody specificity, species compatibility, and potential cross-reactivity are known to affect flow cytometric detection of surface markers, particularly in nonhuman or less-characterized cell sources. Therefore, both biological heterogeneity and methodological considerations should be taken into account when interpreting the phenotypic profile of these cells.

MSCs constitute only a small fraction of the total cell population in their native tissues. Therefore, the isolation of cells that retain their regenerative potential is of critical importance for research purposes (Yuce et al. 2024). In our study, a time-dependent decline in cellular proliferation in cells from both colostrum and mature milk was observed, accompanied by a progressive increase in PDT. This reduction in proliferative capacity may be associated with replicative senescence. This phase, which occurs after the logarithmic growth phase, is characterized by the maintenance of metabolic activity concomitant with a decline in proliferative capacity, and is commonly referred to as the Hayflick limit (Acar 2015; Izadpanah et al. 2008). It has been reported that in umbilical cord-derived MSC lines, the PDT increases significantly with prolonged culture (Koltsova et al. 2018). This finding aligns with the decrease in proliferative capacity observed in our study and suggests that these cells may approach their replicative limits over time.

Our data reveal that PDTs and CFE did not differ significantly between the two groups (*p* > 0.05). This indicates that the fundamental rate of cell division and the frequency of cells with clonogenic potential remained comparable between colostrum and mature milk. In contrast to our findings, Krylova et al. (2012) reported significant differences in PDT values depending on the cell source in their comparison of the proliferation dynamics of embryonic stem cells, fetal bone marrow-derived stem cells, and cells isolated from postnatal foreskin. Moreover, in the same study, the temporal decline in proliferative capacity was interpreted in the context of the Hayflick limit described in the literature (Krylova et al. 2012). Further underscoring the importance of the cellular source, Beeravolu et al. (2017) demonstrated that cells derived from the cord–placenta junction exhibited greater proliferative and self-renewal capacities than those obtained from the umbilical cord, cord membrane, Wharton’s jelly, and fetal placenta (Beeravolu et al. 2017). A study evaluating the clonogenic potential of MSCs in three bone marrow-derived cell populations revealed colony numbers ranging from 12.55 to 152.41 per 10^5^ cells (Kastrinaki et al. 2008). Similarly, CFE values in MSCs isolated from bone marrow have been reported to vary between 3.2 and 7.4 per 10^5^ cells depending on donor and methodological differences (Samsonraj et al. 2015), highlighting the considerable variability of clonogenic assays across experimental conditions. In a study conducted in horses, the CFE of MSCs derived from different regions of subcutaneous tissue, gingiva, and periodontal ligament was also reported to vary significantly (Mensing et al. 2011). Regarding the potential impact of lactation stage and maternal factors, a human study that evaluated CFE in breast milk-derived cells from mothers reported higher CFE on an early postpartum day compared with day 15 and noted a decreasing trend in CFE with advancing maternal age (Nosrati Tirkani et al. 2019). This shows that donor-related factors can influence the potential for clone formation. To the best of our knowledge, our study is the first to evaluate the CFE of colostrum and mature milk-derived cells. In the present study, the mean colony numbers were 1.11 ± 1.82 and 1.39 ± 1.32 for colostrum-derived and mature milk-derived cells, respectively. Taken together, these findings suggest that the low colony counts observed in our study may reflect site-specific variations in the availability of clonogenic cell subpopulations, the influence of donor-related factors such as age and parity, as well as potential differences in the initiation of colony formation by bovine milk-derived cells in vitro.

We employed the MTT assay to evaluate cell viability on the basis of metabolic activity (Tokur and Aksoy 2017). Despite comparable PDT and CFE between colostrum- and mature milk-derived cells, colostrum-derived cells exhibited significantly higher metabolic activity from day 2 onward (*p* < 0.05). This suggests that while the proportion of colony-forming cells and their division speed are similar, colostrum-derived cells exhibit higher metabolic activity.

### Limitations

Although the expression of positive mesenchymal stem cell (MSC) markers (CD73, CD90, and CD105) was confirmed, the analysis of negative hematopoietic and lineage-specific markers (e.g., CD34, CD45, CD14, and HLA-DR) was not performed in the present study. According to the minimal criteria defined by the International Society for Cellular therapy (ISCT), the absence of such negative markers is essential for the definitive identification of MSCs. Therefore, the lack of negative marker profiling limits the ability to conclusively classify the isolated cells as bona fide MSCs and raises the possibility of heterogeneous cell populations. In line with this limitation, the findings of the present study have been interpreted as demonstrating MSC-like properties rather than being definitively classified as MSCs. Future studies incorporating comprehensive immunophenotyping approaches, including both positive and negative marker panels, will be necessary to confirm the precise identity and purity of these cell populations. Additionally, the effects of donor-related factors such as age and parity were not evaluated in the present study.

## Conclusions

This study characterized and compared cells isolated from bovine colostrum and mature milk. Our findings indicate that both sources contain heterogeneous populations with MSC-like properties. Notably, colostrum-derived cells exhibited higher metabolic activity than those from mature milk. However, no significant differences were observed in CFE and PDT values between the groups. These results highlight the potential of bovine milk as a noninvasive and accessible source of MSC-like cells, while also emphasizing the need for further studies to clarify their biological identity, functional capacity, and potential applications.

## Data Availability

All data included in this study are available upon request from the corresponding author.
